# Tracheotomy in Croup

**Published:** 1867-06

**Authors:** Henry Moon

**Affiliations:** Physician to the Sussex County Hospital


*Tracheotomy in Croup.* By Henry Moon, M. D., M. R. C. P. L., Physician to the Sussex County Hospital.

Croup (trachealia, tracheitis, or cynanche trachealis) is a disease of early life, and of sufficient frequency and severity to render it a subject of anxious interest and study to all practising members of the profession. I do not think that we have any statistics of the history and duration of croup when left to the *vis medicatrix naturæ* alone—a wonderful power though it be, and one with which both physicians and surgeons must feel themselves to be but humble co-operators; the power which cures a fever or an inflamed lung as surely as it mends a broken bone or heals a wound. From our present knowledge of the subject, however, most persons who have seen much of croup agree in this, that unless the disease be early recognised and promptly treated, a large proportion of children suffering from it die, under every known system of therapeutics. It is no less true that croup, of all other grave diseases, is often the slowest to be recognised, the early symptoms being regarded by those who have the charge of children as a "feverish cold," and the real importance of the hoarseness, reedy breathing, and the occasional ringing cough, is altogether overlooked for some considerable time.

Is there no method of lessening the mortality in croup? I have long believed that, next in value to an early appreciation and the prompt use of fitting remedies at the onset, *tracheotomy skilfully performed*, and at the *right time*, is a means calculated to save many lives. The right time cannot certainly be determined by days or hours, for the disease sometimes runs a very rapid course, though usually its duration ranges from four to ten days; neither should it be left as a *last resource*, when death from asphyxia appears imminent, and the patient is exhausted from long and fruitless struggles to aërate the lungs, the latter becoming highly congested, inflamed, or œdematous. The *ante-mortem* deposits of fibrine, too, sometimes found after death in the right auricle and ventricle of the heart, judging from the analogy of other instances of fibrinous clots in the cavities of that organ, may mean nothing more than that the patient died a lingering death, with his blood poisoned and his circulation and respiration impeded. In the worst and most unpromising conditions of the disease, however, if tracheotomy fail to save life (and nothing else is so likely even then to succeed,) it will afford great temporary relief; and death from the easy sinking of asthenia bears no comparison in point of suffering with that which results from gradual suffocation.

Whenever, therefore, some signs of amelioration do not follow the steady use of the remedies employed for croup, the powers of the patient becoming less strong, and the predominant symptoms are those of *asphyxia*, whether it be on the second, third, fourth, or sixth day of the disease, *this* is the proper period for the operation of tracheotomy.

I will now venture to copy from my notes the following case:—On the 27th of July last, E. F——, aged six years, a fine, florid, well-nourished boy, lay on wet grass. In the evening he complained of hoarseness, wheezing, and cough. On the 28th, 29th, and 30th the symptoms did not abate, always increasing in severity at night. On the 31st the cough became more harsh, crowing, and ringing in character. The little fellow was ferverish, thirsty, and lost his appetite for food.

Aug. 1st.—E. F—— was brought home from the country to Brighton in the evening; and when I saw him soon afterwards, his skin was dry and hot; his pulse hard and quick: he was feverish and thirsty; the act of inspiration prolonged, accompanied with a reedy piping noise, and an occasional brassy cough, the physical signs of pneumonia or bronchitis being absent, the chest resonant. Severe paroxysms of difficulty of breathing had occurred during his journey. He was covered with a flannel gown, placed in bed in a warm room, the atmosphere of which was rendered moist by a free generation of steam; full emetic doses of antimony and ipecacuanha wines were administered, succeeded by a purgative of calomel and jalap; hot fomentations and poultices to the neck and throat; a milk diet; and twenty minims of antimony wine, with ten minims of the tincture of henbane, every hour.

2d.—He slept soundly at intervals during the night. His skin is less hot and dry; his pulse sharp, but not hard, and less quick; free purging from the bowels; and he is altogether better and more comfortable; yet his breathing is reedy, and, exertion or excitement, the constriction in breathing is increased, and the cough croupal and ringing.

3d.—He passed a restless and sleepless night; the breathing reedy and more laborious; all the muscles of inspiration are brought into action; his chest heaves violently, and the cough is croupal, loud, and ringing; his skin hotter and less moist, and the pulse sharp and quick. Iodide of potassium and senega substituted for antimony wine and henbane.—Afternoon: The boy's powers are not so good; the veins of his neck injected; his face flushed, swollen, and congested; the eyeballs prominent; the lips livid and blue; the chest not quite so resonant, and the breath-sounds at the base of each lung rather coarse. He is drowsy and heavy, and the tendency to death from asphyxia the prominent symptom, which no mere drug agent can prevent; but the admission of air into the lungs, before the powers are further exhausted, is what nature claims, and it is likewise the suggestion of experience and common sense. At half-past five P. M. tracheotomy was most ably performed (under chloroform) by my friend Mr. Nathaniel Blakers, the house-surgeon of the Sussex County Hospital, to whom I am much indebted for his very valuable assistance during the after-treatment. Chloroform did not increase the difficulty of breathing; on the contrary, it allayed spasm in the muscles of the larynx. The first insertion of the canula gave rise to some spasm and distress, but after a short time the breathing was tranquil and the boy much relieved. He takes freely of milk in small quantities.

4th.—He sleeps soundly, and breathes freely and quietly through the tube; he has frequent fits of coughing, and much mucus escapes by the canula. In the afternoon his powers flagged a little, he was restless, and the pulse quickened and was jerking. In addition to the free supply of milk, an ounce of beef-juice, and egg, and fifteen minims of ladanum was thrown into the rectum, to be repeated night and morning; the inner canula to be changed twice a day and cleansed.

5th.—Improved in every way. He slept well. Large pieces of a membranous substance were expelled through the tube by coughing in the night.

6th.—He is cooler, moister, and doing well, and free from signs of pneumonia or bronchitis.

7th.—The boy sleeps soundly, takes his milk eagerly, and amuses himself with his toys.

8th.—The canula removed entirely from the wound in the trachea.

9th.—He breathes well through the nostrils and mouth; he eats light pudding, eggs, bread and milk, fish.

12th.—The wound in the trachea and integuments nearly healed; his voice almost natural.

Sept. 10th.—The cicatrix in the neck is small; the child takes daily exercise out of doors, and is in every respect well in health.

Whether in this instance the obstruction to the passage of air into the lungs were caused by inflammation and the formation of false membrane in the trachea, and spasm of the laryngeal muscles, or to inflammation of the mucous membrane of the larynx, glottis, and trachea, is of comparatively little moment. The condition threatning life wae asphyxia, and tracheotomy was the only remaining remedy.—*London Lancet.*

